# Molecular Characterization of Chelonid Alphaherpesvirus 5 in a Black Turtle (*Chelonia mydas*) Fibropapilloma from Baja California Sur, Mexico

**DOI:** 10.3390/ani11010105

**Published:** 2021-01-07

**Authors:** Eduardo Reséndiz, Helena Fernández-Sanz, José Francisco Domínguez-Contreras, Amelly Hyldaí Ramos-Díaz, Agnese Mancini, Alan A. Zavala-Norzagaray, A. Alonso Aguirre

**Affiliations:** 1 Departamento Académico de Ciencias Marinas y Costeras, Universidad Autónoma de Baja California Sur (UABCS), La Paz 23080, Mexico; 2 Health Assessments in Sea Turtles from BCS, La Paz 23085, Mexico; helena.fdezsanz@gmail.com; 3 Asociación Mexicana de Veterinarios de Tortugas A.C., Xalapa 91050, Mexico; 4 CIMACO, Universidad Autónoma de Baja California Sur (UABCS), La Paz 23080, Mexico; 5 Instituto Politécnico Nacional, CICIMAR, La Paz 23096, Mexico; fradoco@gmail.com; 6 Facultad de Biología, Universidad Veracruzana, Xalapa 91000, Mexico; amelly_3236@hotmail.com; 7 Grupo Tortuguero de las Californias A.C., La Paz 23098, Mexico; agnes@grupotortuguero.org; 8 Instituto Politecnico Nacional, CIIDIR, Guasave 07738, Mexico; anorzaga@gmail.com; 9 Department of Environmental Science and Policy, George Mason University, Fairfax, VA 22030, USA

**Keywords:** *Chelonia mydas*, chelonid alphaherpesvirus 5, fibropapillomatosis, polymerase chain reaction

## Abstract

**Simple Summary:**

Fibropapillomatosis in sea turtles is a neoplastic disease associated with an infection by chelonid alphaherpesvirus 5, which can be fatal to turtles. The Baja California peninsula in the Mexican Pacific has been a relatively pristine environment for local aquatic wildlife; however, in the last decade, several turtles with this disease have been reported in the foraging areas of the region. Reasons for this are unknown but may be related to population growth, the surge of unchecked tourism, pollution, and fisheries, which have increased in the area over the past two decades. Finding a black turtle with fibropapillomatosis and chelonid alphaherpesvirus 5 in a natural protected area to host one of the most important foraging areas for sea turtles in the Mexican Pacific represents a potential risk for the population of black turtles and other species of sea turtles that visit the feeding grounds of the peninsula. This suggests a need to strengthen research lines on the west coast of Mexico and generate conservation strategies for organisms and the ecosystems that they inhabit.

**Abstract:**

During routine monitoring in Ojo de Liebre Lagoon, Mexico, a juvenile black turtle (*Chelonia mydas*) was captured, physically examined, measured, weighed, sampled, and tagged. The turtle showed no clinical signs suggestive of disease. Eleven months later, this turtle was recaptured in the same area, during which one lesion suggestive of fibropapilloma on the neck was identified and sampled for histopathology and molecular analysis. Histopathology revealed hyperkeratosis, epidermal hyperplasia, acanthosis, papillary differentiation and ballooning degeneration of epidermal cells, increased fibroblasts in the dermis, and angiogenesis, among other things. Hematological values were similar to those reported for clinically healthy black turtles and did not show notable changes between the first capture and the recapture; likewise, clinicopathological evaluation did not show structural or functional damage in the turtle’s systems. The chelonid alphaherpesvirus 5 (ChHV5) UL30 gene was amplified and sequenced for phylogeny; Bayesian reconstruction showed a high alignment with the genus *Scutavirus* of the Eastern Pacific group. This is one of the first reports of ChHV5 in a cutaneous fibropapilloma of a black turtle in the Baja California peninsula.

## 1. Introduction

Sea turtle fibropapillomatosis (FP) is a disfiguring, debilitating, and sometimes fatal disease with a complex etiology and pathogenesis that affects sea turtles [1]. The disease has been reported in nearshore regions where human encroachment has led to increases in pollution and habitat alteration resulting in a generally unhealthy aquatic environment for sea turtles [2]. FP is characterized by the presence and development of single and/or multiple tumors (fibropapillomas) that can occur anywhere on the epithelial tissues, and sometimes the carapace/plastron of an affected turtle. Morphologically, FP has a wide variety of appearances based on color, size of tumors, and their anatomical location [3]. Histologically, FP is primarily described as a benign tumoral disease [1] with a molecular characterization associated with the chelonid alphaherpesvirus 5 (ChHV5), a linear double-stranded DNA virus belonging to the *Alpha-herpesvirinae* subfamily and the *Scutavirus* genus [4]. However, FP is a complex disease with a multifactorial etiology. In addition to ChHV5 infection, environmental, microbial, and/or immune-related cofactors most likely influence its pathogenesis [5]. FP was first described in green turtles (*Chelonia mydas*) and it has been referenced in all sea turtle species worldwide in coastal and shallow waters, especially benthic habitats linked to anthropogenic change [2]. Its prevalence has reached epizootic proportions in several populations of sea turtles around the world, causing serious negative effects on individual health and significant impacts at the population level [6]. Severe cases of FP have been associated with clinicopathological abnormalities and metabolic conditions, which together suggest a chronic disease process [7,8,9]. Depending on the location, size, and degree of severity of the tumors, affected turtles often have a series of associated conditions including bacterial, fungal, and/or parasitic co-infections, buoyancy problems, difficulty of predator evasion and boat avoidance, systemic dysfunction, and sometimes, mortality [1]. Until recently, the health status of sea turtles and their infectious diseases in Baja California were poorly understood. After more than 20 years of monitoring the Peninsula, there are only six reported cases of FP in sea turtles, with the first record of FP in a black turtle (*C. mydas*) confirmed by histopathology and transmission electron microscopy in 2010 in San Ignacio Lagoon [10], and the second case reported in a black turtle from Ojo de Liebre Lagoon (LOL) confirmed by gross pathology and histopathology in 2010 (Aguirre and Zavala-Norzagaray, unpubl. data). Since then, there have been four other cases of FP confirmed by histopathology in two olive ridley turtles (*Lepidochelys olivacea*) and one loggerhead turtle (*Caretta caretta*) from the Gulf of Ulloa [11,12], and one in another black turtle from LOL [12]. In the past, the Baja California region has been a relatively pristine environment for local aquatic wildlife, but the surge of unchecked tourism, pollution, and increased fisheries over the past two decades may be a factor related to an increase in the observations of disease prevalence. Since these anthropogenic influences have emerged, sea turtles have been monitored and studied regularly, allowing the identification of possible threats and providing an opportunity to implement effective management strategies. The objective of this study was to provide detailed histopathological description and molecular characterization of ChHV5 in a black turtle fibropapilloma from LOL, Baja California Sur, Mexico.

## 2. Materials and Methods

During routine monthly monitoring in LOL (latitude: 27°55′6.17″, longitude: 114°9′58.30″), a natural protected area within the Biosphere Reserve “El Vizcaíno”, on 20 August 2018, black turtles were captured unharmed using dip nets. For each turtle, a detailed systematic physical examination with craniocaudal orientation was performed by a qualified veterinarian [13,14]; curved carapace length (CCL), straight carapace length (SCL), and mass were recorded [15]. Body condition index (BCI = body mass * 10,000/SCL^3^) was calculated [16]. All turtles were classified based on life stage-class and sex was determined based on external features (CCL and tail length) [17]. Blood samples were obtained aseptically from the dorsal cervical sinuses [18] using 5 mL plastic syringes. The dorsal surface of the neck was disinfected using Germisin^®^ before and after blood collection. The needle (21G × 32 mm) was inserted perpendicularly to the dorsal surface of the neck at a depth of 1–3 cm. The blood samples were carefully mixed with anticoagulant after filling the tubes and were stored in a 6 mL container tube (Vacutainer^®^) with lithium heparin (He/Li) as an anticoagulant for hematological analysis. Immediately, two blood films were prepared for clinicopathological analysis [19]. Finally, the turtles were tagged with Inconel tags [20] after disinfecting using Germisin^®^ and released unharmed at the site of capture.

The turtle with tags 1AS971 (R) and 1AS972 (L) was recaptured on 18 July 2019 in the same area. The recapture presented one lesion suggestive of fibropapilloma on the neck, which was disinfected using Germisin^®^ and collected by infiltrating 3 mL of anesthesia (epinephrine + procaine (Adrecaine^®^ Laboratorios Aranda S.A de C.V. *Reg. SAGARPA Q-0449-093*)), and subcutaneously divided into five points, four around the base of the neoplasms and one in the center. After the application of the anesthesia, the base of the tumor was clamped with hemostasis clamps and the tissue was incised with a No. 20 surgical blade. The lesion was completely resected and divided into two fractions, fixed in 10% buffered formaldehyde and 70% ethanol, and sent for histopathological and molecular analysis respectively in the Autonomous University of Baja California Sur and in BiotechnologikaA2 laboratories.

### 2.1. Hematological Analysis

Hemoglobin was measured using the portable Mission^®^ Hb hemoglobin meter through reflectance photometry. Packed cell volume was measured by capillary tubes centrifuged for 5 min at 15,000 rpm in an automatic micro centrifuge 6k (AS instruments^®^, Veteris, CDMX, Mexico). Red blood cell (RBC) and white blood cell (WBC) counts were performed using the Natt and Herrick methodology [19]. RBC indices (mean cell volume, mean corpuscular hemoglobin, and mean corpuscular hemoglobin concentration) were calculated from the total count of RBCs, packed cell volume, and hemoglobin following the methodology described in [21]. Blood films were stained with a Diff Quick^®^ kit (HYCEL de Mexico S.A. de C.V., CDMX, Mexico), and a 200 WBC differential count of heterophils, lymphocytes, monocytes, eosinophils, and basophils was performed using a Leica^®^ CME microscope (Quiminet, CDMX, Mexico); thrombocytes were reported as adequate, decreased, or increased. RBC (N = 100) and WBC (N = 50) were classified according to morphological characteristics and the clinicopathological interpretation was made as previously described [22].

### 2.2. Molecular Analysis

A tumor sample was analyzed by polymerase chain reaction (PCR) to determine the presence of Herpesvirus infection. This sample was preprocessed for total DNA extraction with the Quick-DNA Miniprep plus kit (Zymo Research, Irvine, CA, USA). The total DNA worked as a template for the PCR reaction for the detection of the DNApol of Herpesvirus that infect turtles. ChHV5 unique long gene UL30 (382 bp) was amplified from the tumor lesion using the UL30For (5′-AGCATCATCCAGGCCCACAATCTG-3′) and UL30Rev (5′-CGGCCAGTTCCGGCGCGTCGACCA-3′) primers [23] and the enzyme GoTaq 1 U (Promega, Madison, WI, USA), under the following amplification conditions: 94 °C 5 min, 1 cycle; 94 °C 1 min, 58 °C 30 s, 72 °C 1 min, 30 cycles; 72 °C 5 min, 1 cycle. The amplification products were visualized with UV light on 2% agarose gel (*w*/*v*) stained with ethidium bromide and purified with the Zymoclean Gel DNA recovery kit (Zymo Research, Irvine, CA, USA), to proceed with Sanger sequencing with the “ABI Big-dye terminator” technique (Thermo Fisher Scientific, Waltham, MA, USA).

### 2.3. Sequence Alignment and Phylogenetic Identification

The UL30 sequences were edited using Chromas Pro Version 1.6 and using MUSCLE multiple alignment tools implemented in Mega 6 [24]. Additionally, 55 GenBank downloaded sequences were aligned with our UL30 sequences using the online software Blastn (https://blast.ncbi.nlm.nih.gov/Blast.cgi). The lowest values admissible for identities and query cover were of 95.10% and 90%, respectively. JmodelTest 2 [25] was used to select the best-fit model of nucleotide substitution for phylogenetic analyses using the Akaike and Bayesian information criteria. The Jukes–Cantor (JC) model was applied for 56 sequences with a Bayesian model implemented in Mr. Bayes Ver 3.2 [26] with 10 × 10^6^ MCMC (Markov Chain Monte Carlo) (×2 run), a burn-in step of 25%, and a sampling frequency each 100 data that were diagnosticated each for 1000 generations. The MCMC converged adequately with a standard deviation below of 0.01, according to Mr. Bayes manual [26]. The phylogenetic trees generated were edited with free software FigTree (http://tree.bio.ed.ac.uk/software/figtree/).

### 2.4. Ethics Statement

This research was conducted with Permits Oficio No. SGPA/DGVS/013214/18 and Oficio No. SGPA/DGVS/12688/19, and all the applicable international, national, and/or institutional guidelines for the care and use of animals were followed.

## 3. Results and Discussion

### 3.1. Captures, Morphometrics, and Blood Values

On 20 August 2018, 18 black turtles were captured in LOL, of which one juvenile was tagged with Inconel metal flipper tags 1AS971 (R) and 1AS972 (L). This individual measured 76.9 cm CCL, 71.1 cm SCL, and weighed 43 kg, presenting good body condition (BCI = 1.19) [16,27]. During its physical exam, the turtle did not show clinical signs suggestive of disease, lesions, or neoplasms, nor barnacles, leeches, or any injury. The turtle’s hematological values are shown in Table 1.

### 3.2. Recapture, Clinical Evaluation, and Gross Pathology

**Figure 1:**
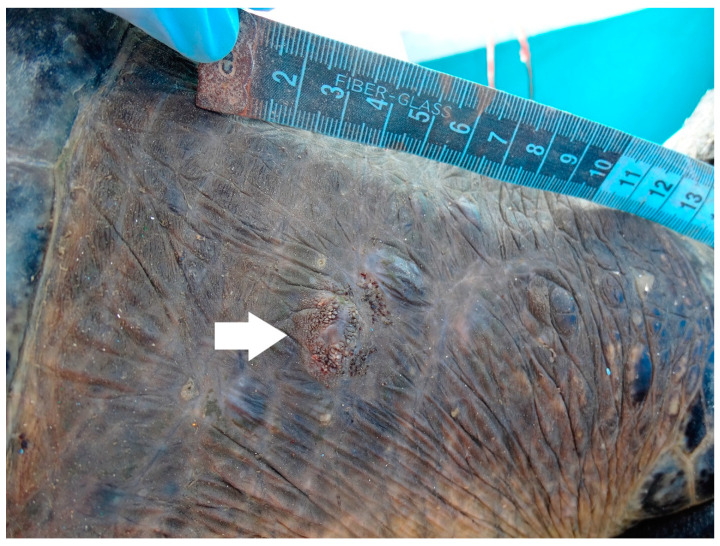
Nodular sessile fibropapilloma of 1.5 cm^3^, with a rough surface, firm consistency, and gray, green, and brown color in the right mediolateral part of the neck of a black turtle (*Chelonia mydas*) in Ojo de Liebre lagoon.

On 18 July 2019, six black turtles were captured in the same area, which included the turtle with tags 1AS971 (R) and 1AS972 (L) (recapture) that was classified as an adult female according to her CCL, 79.6 cm; SCL, 77.5 cm; tail length, 17 cm; and mass, 47 kg [17,28]. The recapture presented a lower body condition (BCI = 1.01) than in first capture. During the second clinical review, a semicircular sessile nodulation of 1.5 cm^3^ was observed in the right mediolateral part of the neck and was identified as a fibropapilloma. It had a rough surface, firm consistency, gray, green, and brown color (Figure 1), and was classified as tumor score 1 (i.e., mildly afflicted) according to proposed scoring criteria [7]. Despite showing this condition, the turtle’s size and mass were consistent with the averages reported for the area [16,29] which suggests that FP in early stages does not have a significant impact on these characteristics [8,30].

### 3.3. Histopathology

**Figure 2:**
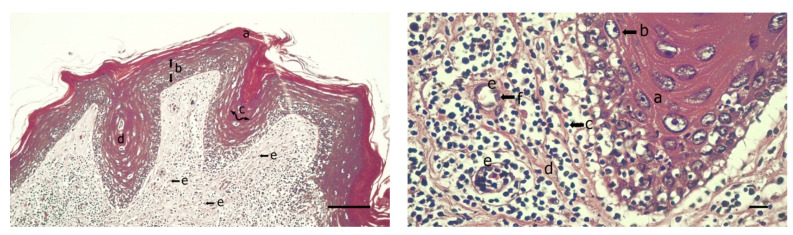
(**Left**) Black turtle (*Chelonia mydas*) cutaneous fibropapilloma. Hematoxylin and eosin. Hyperkeratosis (a), epidermal hyperplasia (b), acanthosis (c), papillary formation supported by a core of fibrovascular stroma (d). Proliferation of fibroblasts in the dermis (e) surrounded by abundant fibrous connective tissue. Scale bar = 100 μm. (**Right**) Black turtle (*Chelonia mydas*) cutaneous fibropapilloma. Hematoxylin and eosin. Papillary projection (a), ballooning degeneration in cells (b), increased fibroblasts in the dermis (c) surrounded by fibrous connective tissue (d), angiogenesis (e) and mild infiltration of lymphocytes (f). Scale bar = 10 μm.

Histopathological findings of the neoplasms revealed orthokeratotic hyperkeratosis, epidermal hyperplasia with areas of edema, acanthosis, dermal papillary differentiation and ballooning degeneration of epidermal cells, fibroblast proliferation in the dermis surrounded by abundant fibrous connective tissue, and moderate lymphocyte infiltration (Figure 2).

These proliferative changes indicated that it was a benign neoplasm and coincided with what was previously reported [1,3,10,12,30]. Among the cytopathic effects related to herpesvirus infection, no intranuclear inclusion bodies were observed. This may be due to the absence of virions during some stages of tumor development [31] or because these are only visible during certain phases of the infection, especially in tumors that already have a longer evolution time [32,33].

### 3.4. Hematology and Clinicopathological Analysis

The hematological values obtained (Table 1) were similar to those previously reported in clinically healthy black turtles in the same area [29] and for green turtles worldwide without FP and with FP score 1 [8,34]. These values did not notably change between the first capture and the recapture and allowed us to rule out a chronic disease process with advanced clinical manifestation in this mild case [7,34]. During the clinicopathological evaluation, no abnormalities or common findings in severe cases of FP were observed (structural or morphological changes and cell derangements) [19], indicating that there was no structural or functional damage detected [22]. Clinicopathological analysis and histopathology indicated that the disease was in the early stages of advancement and the lesion was growing and progressing [1,22].

### 3.5. Molecular Characterization of ChHV5

**Figure 3:**
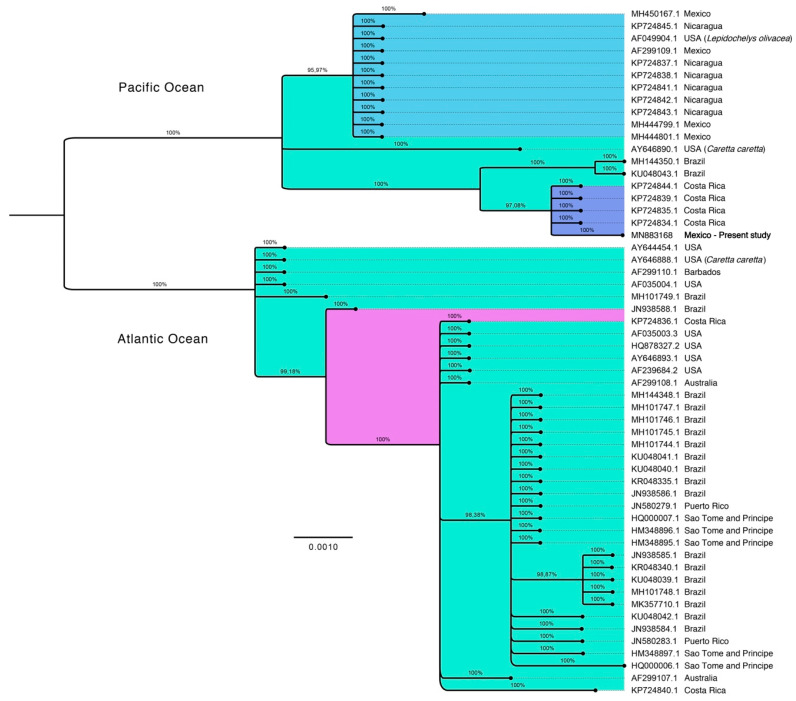
Bayesian phylogenetic tree. UL30 concatenated long sequence of the black turtle (*Chelonia mydas*) from Ojo de Liebre Lagoon and previously reported sequences retrieved from the GenBank. Probabilities are shown for node support. Scale bar indicates nucleotide substitutions per site.

The sample was positive for ChHV5 DNA (GenBank accession number MN883168), and global BLASTN alignment identified that the sequence belonged to the Alpha-herpesvirinae subfamily. Sequence alignment and phylogenetic analysis by Bayesian reconstruction showed the formation of two large phylogenetic groups for the UL30 gene, mainly indicating compound groups for the Atlantic and Pacific Ocean. The phylogenetic relationship for the sequence MN883168 with the genus *Scutavirus* of the Eastern Pacific group (USA, Mexico, Costa Rica, and Nicaragua) was determined, which particularly showed strong alignment with the sequences recorded for Costa Rica (KP724844.1 KP724839.1, KP724835.1, KP724834.1), while the previously reported sequences for Mexico (MH450167.1, AF299109.1, MH444799.1 and MH444801.1) showed a greater relationship with Nicaragua sequences (Figure 3. This is consistent with previous studies that suggest that FP variants are homogeneous between nearby areas [35].

In sea turtles, ChHV5 is suggested to be the primary etiologic agent of FP based on three lines of evidence. First, when tumors analyzed by PCR produce ChHV5 sequences [36]; second, when tumors occasionally show epidermal viral inclusions with ultrastructures similar to those of Herpesviruses [37]; and finally, the experimental transmission of the disease using tumor extracts inoculated in healthy turtles resulting in the development of FP [1,3]. However, elucidating the etiology, pathogenesis, and epidemiology of ChHV5 as a causative agent of FP still remains unfinished, as defined in Koch’s postulates, in addition to other factors that complicate its study and limit the demonstration of its true participation in the development of neoplasms [1]. Despite the fact that only one fibropapilloma classified as tumor score 1 (mild) was studied, the presence of this disease and ChHV5 in a natural protected area that is considered one of the most important sea turtle foraging areas of the Mexican Pacific for black turtles [38] could represent a potential threat to the population of turtles that visit the feeding grounds of the peninsula.

## 4. Conclusions

This is one of the first reports of ChHV5 in a black turtle fibropapilloma in the Baja California Peninsula. The tumor and the cytopathic effects described by histopathology were associated with the presence of ChHV5. The presence of ChHV5 in the Baja California Peninsula represents a potential risk for the populations of sea turtles that inhabit this region, since FP is increasingly frequent at these feeding grounds. Further antibody detection studies (ELISA) are required to confirm ChHV5 exposure and its infectious potential. Additionally, it is proposed to carry out qPCR and ELISA in turtles with and without tumors in order to offer more insight of the ChHV5 status and determine its prevalence in the area. Monitoring sea turtles in foraging areas of Mexico and environmental and anthropogenic factors that promote the dissemination of FP should be intensified to generate reliable information to increase our understanding of the etiology, pathogenesis, and epidemiology of the disease. This information will complement management plans and conservation strategies for organisms and the ecosystem together with local authorities.

## Figures and Tables

**Table 1:** Hematological values of the black turtle (*Chelonia mydas*) from first capture and recapture (eleven months after) when the turtle presented fibropapillomatosis (FP).

Analyte	1st Capture	Recapture with FP
Packed cell volume (L/L)	0.35	0.34
Red blood cells (10^12^/L)	0.58	0.52
Hemoglobin (g/L)	120	118
Mean cell volume (fl)	603.45	653.85
Mean corpuscular hemoglobin (pg)	206.89	226.92
Mean corpuscular hemoglobin concentration (g/L)	342.9	347.1
White blood cells (10^9^/L)	6.0	6.5
Heterophils (10^9^/L)	4.20	3.92
Lymphocytes(10^9^/L)	1.14	1.48
Monocytes (10^9^/L)	0.12	0.29
Eosinophils (10^9^/L)	0.54	0.81
Basophils (10^9^/L)	0	0
Thrombocytes	Adequate	Adequate

## Data Availability

The data presented in this study are available on request from the corresponding authors.
